# Characterization of *prickle* isoform-specific *pk ^pk1^* and *pk ^sple1^* mutations in *Drosophila melanogaster*

**DOI:** 10.17912/micropub.biology.000656

**Published:** 2022-10-20

**Authors:** Anthony J Lilienthal, Mrutyunjaya Parida, J Robert Manak

**Affiliations:** 1 Dept of Biology, University of Iowa; 2 Dept of Pediatrics, University of Iowa, Iowa City, IA, USA

## Abstract

We used paired-end next generation sequencing (NGS) to characterize the classic isoform-specific *pk ^pk1^* and *pk ^sple1^* mutations of the *prickle* gene in *Drosophila melanogaster* . Here we provide evidence that these previously reported null mutations are caused by either a *tirant* transposon insertion into the 5’ UTR of *pk ^pk1^* or a premature stop codon in the second exon of *pk ^sple1^ .* Additional likely benign missense mutations were identified in both mutant isoforms.

**Figure 1. Mapping of exonic alterations in pk pk1 and pk sple1 Drosophila mutants.**
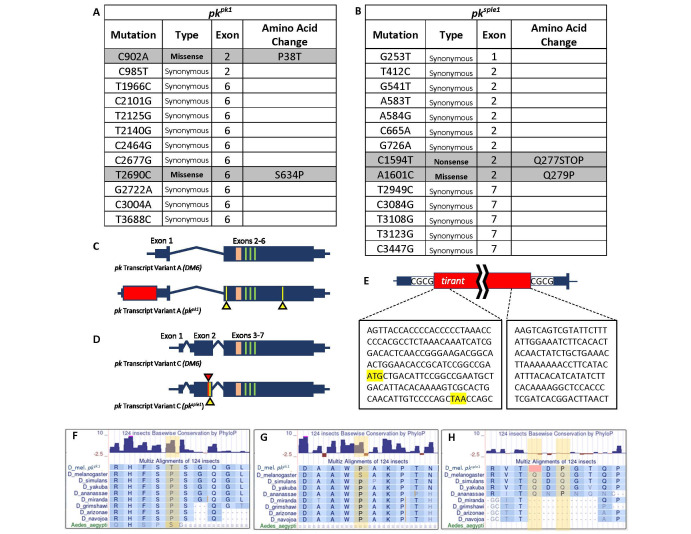
**(A)** Table describing all missense mutations identified in exons of *pk transcript variant A* ( *pk ^pk^* ) for the *pk ^pk1^* mutant. Mutation locations are indicated by number in relation to the reported mRNA sequence. Both exons indicated (2 and 6) are shared between isoforms. **(B)** Table describing all mutations in the exons of *pk transcript variant C (pk ^sple^ )* for the *pk ^sple1^* mutant. Mutation locations are indicated by number in relation to the reported mRNA sequence. Exons include the *pk ^sple^* isoform-specific exons (1 and 2) as well as the shared exon 7. **(C)** Graphical representation showing alterations in *pk transcript variant A* for the *pk ^pk1^* mutant. The red box in the 5’ UTR region depicts the *tirant* insertion, while the yellow lines and arrowheads indicate the missense mutation sites described in panel A in relation to the PET (pink) and LIM (green) domains. **(D)** Graphical representation showing alterations in *pk transcript variant C* for the *pk ^sple1^* mutant. The red and yellow lines and arrowheads indicate nonsense and missense mutations, respectively (exon 2). **(E)** Magnified view of the *tirant* insertion site in *pk transcript variant A* for *pk ^pk1^* . The boxes below the *tirant* schematic show an enlarged view of the PCR-confirmed DNA sequences adjacent to and within the transposon insertion with yellow boxes highlighting the first translation start codon along with in-frame stop codon. A duplication of the CGCG region normally found at the insertion position is indicated in the 5’ UTR flanking the *tirant* sequences *.* **(F – H)** Comparison of amino acid sequence among several *Drosophilidae* family members in addition to mosquito ( *Aedes aegypti* ) in region of the missense and nonsense mutations of *pk ^pk1^* and *pk ^sple1^* .

## Description

The *prickle* gene in *Drosophila melanogaster* has been studied for decades for its involvement in planar cell polarity (PCP), and more recently, epilepsy (Tao et al. 2011, Matis and Axelrod 2013, Ehaideb et al. 2014, Ehaideb et al. 2016). *prickle* expresses three isoforms, *prickle-prickle* ( *pk ^pk^* ; pk-RA, FBtr0089042), *prickle-M* ( *pk ^M^* ; pk-RB, FBtr0089043) *,* and *prickle-spiney-legs (pk ^sple^* ; pk-RC, FBtr0089044 *)* . While *pk ^M^* expression is initiated during embryogenesis, expression ceases prior to the end of pupation (Gubb et al. 1999). On the other hand, both *pk ^pk^* and *pk ^sple^* are only expressed post-embryonically (Gubb et al. 1999). All isoforms arise from alternative start sites and differ based on 5’ coding or non-coding exons (Gubb et al. 1999). Mutations that affect *pk ^pk^* or *pk ^sple^* isoforms have been shown to alter development of the fruit fly body plan including the orientation of eye ommatidia as well as positioning of epidermal hairs and bristles of the wing and body (Gubb 1998, Gubb et al. 1999, Green et al. 2000, Strutt and Strutt 2007, Lin and Gubb 2009, Jenny 2010, Matis and Axelrod 2013, Carvajal-Gonzales and Mlodzik 2014). In addition, *pk* isoforms are required in the nervous system to ensure proper neuronal wiring and function (Mrksusich et al. 2011, Ng 2012, Ehaideb et al. 2014), while mutations specifically affecting *pk ^M^* have not been identified (Gubb et al. 1999). Although some mutations affecting *Drosophila prickle* isoforms have been approximately defined ( *pk ^pk-^* ^sple13^ ; Gubb et al. 1999) or mapped ( *pk ^pk30^* ; Green et al. 2000), the original *pk ^sple1^* and *pk ^pk1^* mutations (Gubb and García-Bellido 1982) have heretofore remained uncharacterized.

We used paired-end Illumina next generation sequencing to characterize and map *pk ^pk1^* and *pk ^sple1^* mutations. On average, for the *pk ^pk1^* (and *pk ^sple1^* ) mutants, 96.5% (and 96.8%, respectively) of the genome had at least 1X read coverage, 92.3% (and 93%, respectively) had at least 10X read coverage, and 84.3% (and 85.8%, respectively) had at least 30X read coverage. For the genomic region encompassing the *prickle* locus, both mutants had at least 30X read coverage. No mutations affecting predicted splice-sites were found in either mutant. Two nonsynonymous mutations, C902A and T2690C, were identified in *pk ^pk1^* (Figure 1A), neither of which fall within the evolutionarily conserved protein-protein-interaction PET and LIM domains (Figure 1C; Gubb et al. 1999, Sweede et al. 2008). Additionally, alignment of paired-end reads to the dm6 *D. melanogaster* genome revealed a region 13 bases downstream of the transcription start site of *prickle transcript variant A* that only showed alignment of one out of the two reads of numerous paired-end sequences *.* Analysis of the unmapped read of these pairs identified sequences from a *tirant* transposon (Figure 1C; Cañizares et al. 2000) which was confirmed with PCR followed by Sanger sequencing (partial sequence for the *tirant* insertion is shown in Figure 1E). This transposon insertion is thus likely responsible for the loss-of-function phenotype in the *pk ^pk1^* mutant, particularly given the numerous start and stop codons at the 5’ end of the *tirant* sequence which would be predicted to be utilized in favor of the correct distal downstream *pk ^pk^* start codon (see Figure 1E). In *pk ^sple1^* homozygous mutants, we identified nonsense (C1593T) and missense (A1601C) mutations in the coding sequence, both of which were located at the 3’ end of the *pk ^sple^* -specific second exon of *prickle transcript variant C* and upstream of the PET and LIM domain exons (Figures 1B, 1D). These data suggest that the premature stop codon is likely responsible for loss-of-function of *pk ^sple^* in the *pk ^sple1^* mutant.

In order to assess whether the nonsynonymous mutations found in the *pk ^pk1^* or *pk ^sple1^* sequences could significantly affect *prickle* gene function, we examined the conservation of amino acids in these regions. In *pk ^pk1^* , while both mutations fall in conserved regions (as predicted by PhyloP in the UCSC Genome Browser; Figures 1F and 1G), they are likely tolerated and non-deleterious. C902A of *pk ^pk1^* (Figure 1F) replaces proline with threonine, an amino acid that is similar in structure to the serine found at that position in a close outgroup to the *Drosophilidae* family ( *Aedes aegypti,* the mosquito), although this region of the protein shows relatively poor conservation between *D. melanogaster* and *A. aegypti* . T2690C also is likely non-deleterious, as *Drosophila melanogaster* is the only member of family *Drosophilidae* shown to contain a serine at this location while all others carry the amino acid that is inserted due to the missense mutation (proline; Figure 1G). Further evidence is found in *D. melanogaster* sequences submitted to GenBank ( www.ncbi.nlm.nih.gov/genbank/ ) that indicate a proline at this position (GenBank identifiers AJ243708.3, BT126167.1). In *pk ^sple1^* , the nonsynonymous mutation occurs immediately downstream from the premature stop site and replaces a glutamine with proline, the amino acid found at the analogous position in another member of the *Drosophilidae* , *D. ananassae* (Figure 1H). Additionally, this region of *pk ^sple^* is poorly conserved. Collectively, these results argue that the missense mutations found in *pk ^pk1^* and *pk ^sple1^* are less likely to be deleterious.

## Methods

Outcrossing of *pk* Lines

Both *pk ^pk1^* and *pk ^sple1^* were outcrossed for 10 generations into a *w ^1118^* line obtained from Dr. Andy Frank (University of Iowa) prior to whole genome sequencing. This line was chosen because it has robust neurotransmission and growth properties at the neuromuscular junction (Yeates et al. 2017). *prickle* mutant bristle phenotypes were confirmed at each relevant step.

Whole Genome Sequencing :

Genomic DNA (from 5 whole male and 5 whole female flies) was extracted with the DNeasy Blood & Tissue Kit (Qiagen). Library preparations and whole Genome Sequencing (paired-end 2 x 150 bp reads, Illumina NovaSeq 6000) was performed by the Iowa Institute of Human Genetics (IIHG) Genomics Division at the University of Iowa (https://medicine.uiowa.edu/humangenetics/genomics-division).

Genome Assembly

FastQC program v0.10.0 (Wingett and Andrews 2018) was used to perform quality control analysis of the paired end sequencing data for the *pk ^pk1^* and *pk ^sple1^* samples. Illumina adapter contaminants were removed from the sequencing data using the Trimmomatic v0.32 (Bolger et al. 2014) with the following settings ILLUMINACLIP: <file of ADAPTERS.txt> :4:40:12 HADCROP:15 SLIDINGWINDOW:5:30 AVGQUAL:30 CROP:130 MINLEN:36. Both single and paired end reads retained after adapter trimming were mapped to the dm6 genome using bwa v0.7.5 (Li and Durbin 2009) and samtools v0.1.18 (Li et al. 2009). Reads with one pair mapping to a given genomic interval in chr2R of the dm6 genome and their associated unmapped pair were identified using a bedtools v2.26 (Quinlan et al. 2010), samtools v1.31 (Li et al. 2009), awk programming language, and bash scripting. This data was used to identify the insertion of a transposon sequence in the *pk ^pk1^* mutants in the neighboring region of these mapped single end reads.

Total reads were counted from the fastq.gz files of every sample using Linux shell commands. Trimmed read and mapped read counts were generated from .bam alignment files of every sample using samtools flagstat command. Total bases of the dm6 genome were computed from the chromosome sizes file that was generated from the FASTA index file of the dm6 genome using samtools v0.1.18 (Li et al. 2009) and Linux shell commands. The chrUn and chromosomes with ‘random’ in their sequence identifiers were filtered out. Coverage for every sample was generated by counting the number of bases in the dm6 genome covered by at least 1, 10, or 30 mapped reads independently using bedtools v2.26 (Quinlan et al. 2010) genomcov program and shell scripting.

Sanger Sequencing using an ABI3500 Sanger Sequencer was performed on PCR-amplified fragments to confirm both the insertion of the *tirant* transposon of *pk ^pk1^* and the premature stop codon of *pk ^sple1^* mutants as well as all other missense mutations. The Multiz Alignment tool (Blanchette et al. 2004) in the USCS Genome Browser (Kent et al. 2002, Karolchik et al. 2004) was used to compare amino acid sequences.

## Reagents

**Table:** 

*Drosophila Melanogaster*			
Stock Number, Bloomington *Drosophila* Stock Center	Genes Affected	Genotype	
BDSC 367, FBst0000367	*w, pk, cn*	*w[1118];pk[1] cn[1]*	
BDSC 422, FBst0000422	*w, pk*	*w[1118];pk[sple-1]*	
n/a; line obtained from Dr. Andy Frank	*w*	*w[1118]*	
